# What is the Meaning of Paid Employment for Well-Being? A Focus Group Study on Differences and Similarities Between Autistic Adults With and Without Employment

**DOI:** 10.1007/s10926-023-10136-0

**Published:** 2023-09-25

**Authors:** Evelien P. M. Brouwers, Michel Bergijk, Jaap van Weeghel, Sarah Detaille, Jeroen Dewinter

**Affiliations:** 1 Tranzo Scientific Center for Care and Wellbeing, Tilburg University, Tilburg, The Netherlands; 2 HAN University of Applied Sciences, Arnhem, The Netherlands; 3 GGzE Centre for Child and Adolescent Psychiatry, Eindhoven, The Netherlands; 4 Tilburg School for Social and Behavioral Sciences, Tranzo Scientific Center for Care and Wellbeing, Tilburg University, PO Box 90153, 5000 LE Tilburg, The Netherlands

**Keywords:** Autism, Well-being, Employment, Meaning, Mental health, Autistic burnout

## Abstract

**Purpose:**

The aim of the present study was to explore the meaning of work for the subjective well-being of autistic adults with and without paid (competitive) employment and to evaluate the differences and similarities between these groups.

**Methods:**

Eight focus groups were conducted, including a total of 64 autistic adults. Four groups entailed participants *with* current paid employment (including part-time) and four groups entailed participants *without paid employment*. All discussions were audiotaped and transcribed verbatim to enable inductive thematic content analysis. Data were analyzed using ATLAS.ti 9.

**Results:**

Generally, both groups viewed paid employment as very important for well-being, albeit for different reasons. Three themes were found: (1) Not having paid employment was associated with lacking societal recognition, and subsequent low self-esteem, which was a dominant theme in those *without* work; (2) Work can seriously damage (mental) health and well-being*,* found in *both* groups; and (3) Paid employment provides many benefits for well-being*,* with subthemes: *‘purpose,’ ‘social contacts,’ ‘growth and use of talents,’ ‘structure and calmness,’ and ‘income and freedom’*, which was a dominant theme in those *with* paid employment.

**Conclusions:**

Both groups found paid employment highly important for their well-being, albeit for different reasons. However, both also agreed that paid employment can be very harmful to (mental) health and well-being. Suitable, well-supported jobs are important for well-being, may help to buffer stress in other life areas, and may even prevent autistic burnout. More studies are needed on how healthy jobs can be created where autistic individuals get positive energy and experience high well-being. This will also help to reduce socio-economic inequality.

**Supplementary Information:**

The online version contains supplementary material available at 10.1007/s10926-023-10136-0.

## Introduction

Employment rates of autistic people^1^ remain poor. For instance, a recent report from the Office for National Statistics in the UK showed the employment rate of autistic adults to be only 29% in 2021 [3]. In the Netherlands Autism Register, a large cohort of autistic people, only 48% of participants reported to have paid employment in 2021 [4] compared to 72.2% of the general population [5]. These lower employment rates of autistic people are problematic for a variety of reasons. First, employment can provide major benefits for mental health and well-being [6–8], such as daytime structure, financial independence, self-confidence, and autonomy [9, 10]. Also, unemployment and job loss have shown to impair mental health [11], whereas re-employment after unemployment has been shown to lead to increased health [6, 7]. A second reason why the low employment rates of autistic adults are problematic, is for economic reasons. Unemployment is associated with poverty [12], and even if autistic individuals receive social or disability benefits, this often means their income is low. For society, the costs of lost productivity and social and disability benefits are also troublesome [12]. A systematic review on the costs and benefits of employing autistic adults concluded that enhancing the opportunities for autistic adults to join the workforce is not only beneficial from a societal and inclusiveness viewpoint, but also from a strict economic standpoint [13]. The current and future shortage of workers due to an aging working population highlight the importance of improving employment rates of autistic adults even further.

In the scientific literature on sustainable employment, an important transition in thinking has occurred in the past two decades, providing new directions for improvement. To be specific, previously the medical model prevailed, viewing ‘disease’ as the primary reason for lack of employment, requiring the individual to take responsibility for their disability and make the necessary personal adjustments to be eligible for employment [13, 14]. Consequently, so far interventions have mainly been individual and impairment- focused, trying to ‘fix’ the autistic adult [13], overlooking the role of the work environment, as well as strengths, talents and preferences. Currently, attention for the role of the work context is growing, as is the field of ‘positive psychology,’ focusing on people’s strengths and talents and the positive aspects of human functioning rather than on pathology [15]. Studies have shown that interventions focusing on what individuals find really important for their well-being and how they can realize this at work are often highly successful for enhancing sustainable employability (e.g., see papers on the Capability approach [16, 17], Individual Placement and support [18], Job crafting [19, 20], and Job Demands-Resources theory [21].

The extent to which people find having paid work essential for their subjective well-being, and *what* aspects they find important, may depend on their experiences with finding and keeping paid employment. For instance, negative experiences such as becoming unemployed, or job insecurity (i.e., expecting involuntary job loss) have shown to leave ‘scarring effects’ on peoples’ long-term health and well-being [22, 23]. Moreover, a study on the meaning of work for work disabled people showed that meanings and values ascribed to work often change following disability. New meanings, found either at home or in modified work, can replace the old and contribute to new identities [24]. Although several studies have evaluated autistic adults’ work motivation and experiences, [10, 25], few have considered that the meaning of work may differ between those who do and do not have paid employment. Therefore, the aim of the current study was to explore the meaning of work for autistic adults with and without competitive employment. Specifically, research questions were:

1. What do autistic adults believe is the meaning of paid employment for their subjective well-being?
2. What are differences and similarities between autistic adults *with* and *without* paid employment, in what having paid employment means for their subjective well-being?

## Methods

In November and December 2019, eight focus groups were conducted, including 64 autistic adults in total. Group size ranged from 4 to 13 participants. There were two types of focus groups: (1) four focus groups with participants *who had paid employment at the time of the focus group* (*N* = 40, including part-time), and (2) four focus groups with participants *who did not have paid employment at that moment* (*N* = 24). There were no repeat interviews, i.e., participants could only take part in one group discussion. Total duration of the meetings was two hours, of which the first 30–60 min were used for this study, and the second part for a separate study on barriers and facilitators for sustainable employment (not further reported in this paper). Participants received a gift certificate of 10 euros and travel costs were reimbursed. Findings are presented following the *Consolidated criteria for reporting qualitative studies* (CORECQ) [26]. See appendix A for the completed checklist.

### Participants

Convenience sampling [27] was used to recruit eligible participants. Permission was obtained to recruit at the annual Dutch ‘Autminds’ 2019 conference in Amsterdam. Also, members of the Dutch autism association (NVA) were contacted, as well as work reintegration specialists from a large mental health care institute. Moreover, occupational health specialists (e.g., occupational physicians) and other members of the personal network of the researchers were either contacted in person, or through Twitter and LinkedIn. Participants were eligible if they reported to have received a formal Autism Spectrum Disorder diagnosis, provided by a mental health care professional (e.g., psychologist, psychiatrist). A total of 27 people declined participation after receiving more information about the study. Reasons to decline were as follows: being unavailable at the times and dates of the focus groups (*N* = 10), traveling to the focus group location was too far or would cost too much energy (*N* = 6), no interest in the study (*N* = 9), and did not show up (*N* = 2).

Of the total group of 64 participants, 28 were single, 36 were female (vs 27 males and one other), and participants were rather highly educated, with 39 having an (applied) university degree. All participants were Dutch Caucasians. The mean age was 47 (SD 10.98). Those with paid employment worked in different professions, e.g., as a school teacher, lawyer, cleaner, assistant pharmacist, and systems engineer. Their mean number of hours of paid employment per week was 31.2 (SD 8.6).

### Focus Group Meetings

All focus group meetings were audiotaped and transcribed verbatim, to enable inductive thematic content analysis [27]. The meetings took place at conference rooms at three different locations in The Netherlands: at Tilburg University, at a mental health knowledge institute in Utrecht, and at a mental health care organization in Eindhoven. All groups were guided by EB (female professor), or JD (male senior researcher), both PhD, educated and experienced in guiding focus groups for scientific research. MB, a male junior researcher (MSc) was present at all meetings and had the role of observer. During one focus group, a personal coach was present at one participants’ request, but did not participate in the conversation. Field notes were made during and after the focus groups.

At the start of each focus group, the researchers introduced themselves, informing participants that JD also worked as a clinical psychologist with autistic youth, and that MB was an autistic researcher. After the introductions, the goals of the study were explained, and questions were answered. All focus groups started with the general question to what extent participants found having paid employment important for their subjective well-being. The topics discussed were not pilot tested. After eight focus groups, no new information was gathered, and data saturation was reached. After the focus groups, participants were not contacted again for a member check or feedback on the literally transcribed audio recordings. The topic list can be found in Table 1.

**Table 1:** Topic list

To what extent is having paid employment important for your well-being? For what reasons is having paid employment to some extent important (or not) for your well-being? Do others recognize these reasons? What is it like for them? Can you think of an ideal job? If so, what would such a dream job look like to you, and why?	
Are there any particular aspects of paid employment that you do or do not find (very) important for your well-being? Can you explain why? If you do have experiences with paid employment, how have they affected your well-being?	
What experiences do you have with paid employment?	
What are any additional thoughts you have on the meaning of paid employment for your subjective well-being?	

### Coding, Data Analysis, and Interpretation of the Data

All transcripts were anonymized before analyses were performed. To increase reliability, each transcript was read repeatedly, and coded by 2–3 researchers independently. Codes were created using open, axial, and selective coding [28]. In line with the constant comparison principle, codes were compared and the relationships between codes were explored to find themes and subthemes [27]. This process was done by EB who clustered the codes and defined emerging themes. Next, these results were reflected upon and discussed by between EB, MB, JD, and SD until consensus was derived on the interpretation of the themes. Most of the discussions and reflections were done by EB, MB and JD. The analyses were conducted separately for the groups with paid employment and without, in order to explore differences and similarities between the two types of groups. If comparable (sub-)themes emerged, the same theme titles (e.g., “Paid employment can damage health”) were used to enhance visibility of similarities and differences. Data were analyzed using the software program ATLAS.ti version 9.

### Ethical Considerations

Prior to the study, the study proposal was reviewed and approved by the Ethics Review Board of Tilburg University (number EC 2019.68). All participants were informed verbally and in writing about the study before signing a written informed consent form.

## Results

### Theme 1: Not Having Paid Employment was Associated with Lacking Societal Recognition, and Low Self-Esteem

A difference between the two groups was that this was a very strong theme in those without paid employment. In the groups *with* employment, this theme was also discussed, but much less elaborately. Without paid employment, participants missed societal recognition, which was associated with feelings of low self-esteem. Even if they had volunteer work, the absence of *paid* work made them feel that others valued them as less important. Many believed that by not earning their own income, they were not really being taken seriously by others. This had a very strong negative effect on their self-worth and well-being, and participants often reported to feel guilty, ashamed, and socially excluded.

“ …Not having a job feels like [..] not being part of it, feeling low self-esteem [..] failing in the roles I should fullfill in society.”

*(participant without paid employment)*

“…it feels like you are dangling on the outside of society […] I really feel I like I am falling short.”

*(participant without paid employment)*

“Seven years ago, I got burned-out, and now I do volunteer work. I try to fill my days with meaningful activities, but I miss the recognition from society. Like you’re not really part of it if you don’t have paid employment“.

*(participant without paid employment)*

In the groups without paid employment, several participants experienced social isolation. Some felt so looked down upon by others, or so self-conscious about not being able to earn their own income, that they preferred to avoid contacts with others.

“When I lost my job, I noticed that [..] if you don’t work, […] birthday parties are not enjoyable anymore, because they will ask: how is your job? What do you do? Well, I don’t do anything.”

*(participant without paid employment)*

“I don’t want to go shopping anymore, because the neighbors [will] think I am at home all the time [and judge me for it].”

*(participant without paid employment)*

### Theme 2: Work Can Seriously Damage Health and Well-Being

A similarity between the groups was that most participants stressed the importance of paid employment for their well-being. However, also in both groups, many said they had had very damaging experiences through work.

“I would not want to miss it [my work], but on the other hand it gives me so much stress that I believe that as a human, I do not function optimally.”

*(participant with paid employment)*

Very high stress levels were common. Not recognizing or respecting their own (energy) boundaries had left many feel depleted.

”I have worked fulltime. I kept going for sixteen years, but it [..] cost me so much energy that in fact I was only working and sleeping. I just did not have any room for other things anymore”

*(participant without paid employment)*

In both groups, many participants reported to have experienced one or multiple burnout episodes during their lives. In the groups without employment, the severe health damage resulting from previous employment experiences discouraged participants to go back to work, although even then they often could still reflect on what their ideal job would look like. Especially in the groups without work, some participants indicated that their health problems had long lasting, chronic effects on their lives.

“*I got burned out first, and then I got really ill […Now] I feel like I am broken [..]. My body and brain just don’t feel good [..] I cannot think clearly anymore. Physically I am not fit either [..]. I have gone too far now. A burnout too far, a depression too far, an anxiety, and now I am broken.”*

*(participant without paid employment)*

### Theme 3: Paid Employment Provides a Variety of Major Benefits for Well-Being

In both groups, most participants stressed the importance of paid employment for their well-being and mental health. For example, it made them feel useful, gave them a sense of belonging, provided income, allowed them to use their talents and get recognition. It generally made them feel good. A difference between groups with and without work, was that this theme was discussed much more elaborately in the group with employment than in those without. Especially five subthemes highlighted the major advantages that work can have for well-being and mental health: meaningfulness, social contacts, personal growth, structure, and income.

#### Subtheme 1: Doing Meaningful Things and Feeling Useful is Important for Well-Being

A prominent subtheme illustrating the advantages of paid employment for well-being, was purpose: doing meaningful things and feeling useful. Several people commented on the fact that they found meaningful work more important than a high salary. Participants wanted to contribute to something valuable to themselves, to others, or to society. Conversely, not having paid employment made them feel useless, because they did not feel they added value to others or to society.

“I cannot provide myself with sufficient purpose when I am at home. I simply need […] the feeling that I can spend my time in a meaningful way.”

*(participant with paid employment)*

“[Paid] work is important to me because I want to achieve something. I am a product developer, so I want to make a product that sells.”

*(participant with paid employment)*

#### Subtheme 2: Paid Employment as Positive Source of Social Contacts

Many participants mentioned that employment provided them with the opportunity to have positive social contacts and to be appreciated by others, which participants found highly important for their well-being. Employment was also seen as a protective factor against social isolation and loneliness.

“I find it [my job] enjoyable. It is fun, I have nice coworkers […], a lot of fun. Yeah, I don’t have such fun at home.”

*(participant with paid employment)*

“During the past years I was forced to be at home, and after a while I noticed that I am now deteriorating, cognitively, because I don’t have any sparring partners, and don’t have anyone to talk to.”

*(participant without paid employment)*

Interestingly, several participants stressed that work was important for their well-being, because the social contacts they had at work were less demanding and difficult than those in their private lives:

“Friday nights I already look forward to Monday mornings. It is not that I dislike spending time with my family […], but I know that by the end of the weekend so many things have gone wrong, things I have said wrong, where I have expressed myself in a clumsy way, that I think: when I start on Monday there is no one who will be difficult the way they are at home. [...] I am sure people at work will think I am weird or have crazy ideas, but they will not express themselves as much […]. The energy drain at home was because I didn’t feel appreciated, whereas the energy boost I got at work was because I felt I *was* being appreciated. [..] at work, I do not have to deal with all sorts of social emotional topics.”

*(participant with paid employment)*

“In the beginning of my career [..] I used my work as a hiding place because I could not handle having a family with three young children [..]. So I fled to the office and came home around ten, eleven in the evening, when I knew for sure [..] my children were in bed.”

*(participant with paid employment)*

#### Subtheme 3: Personal Growth and Use of Talents are Important for Well-Being

Many participants spoke positively of paid employment as an opportunity for personal growth and use of their talents.

“I am talking about personal growth. Your brain needs to stay active, you need to learn new things[..] If there were any problems at work, I [used to like to] think about where they came from and what we could do about them [..] Yeah wonderful.”

*(participant without paid employment)*

“For me it is a place where I can grow and use my talents. And that is more important to me than the financial aspect.”

*(participant with paid employment).*

#### Subtheme 4: Structure and Calmness are Important for Well-Being

Most participants, especially in the groups with paid employment, said that paid employment brought structure and rhythm in their lives. This in turn brought peace and calmness, which they regarded as highly important for their well-being and mental health.

“If I do nothing, I get bored and go crazy. [Work] keeps me calm and keeps me going. [..] and helps me to get through the week [..]. If I don’t have it, things will get really, really difficult for me.”

*(participant with paid employment)*

“…I am actually extremely happy [..] that I can work again, simply because I need structure in my life. It [work] simply is something to get up for in the morning, to brush my teeth for, to take a shower […] if I am at home all the time […] I cook meals less often, I don’t put much effort into my personal hygiene. Structure really helps a lot, like from work or school.”

*(participant with paid employment)*

#### Subtheme 5: Income and Freedom

In both groups, participants indicated that earning ones’ own income was highly important for well-being. Earning their own income not only meant having money but was also associated with feelings of pride and independence.

“ I want to keep control over my own independence. If I don’t have work I am dependent on others who will determine what my life will look like.”

*(participant with paid employment)*

“ Not having to beg others if you want to buy something small for yourself, because that is actually very humiliating. If only you earn a little bit of money that you can spend, you already feel more proud of yourself [..] because you are an adult, and not a child anymore. If you don’t have any money at all, you feel a bit like a child.”

*(participant without paid employment).*

In the groups without paid employment, several participants received long-term disability benefits. This greatly reduced their stress, as it ensured them a safe source of income, reduced financial stress and relieved them from the pressure of seeking and maintaining employment. Also, they were relieved they no longer had to engage in the lengthy, stressful and energy consuming application procedures to be granted disability benefits. Despite this relief, when asked if they could imagine what their ideal job would be like, many could still describe such a dream job. Finally being granted disability benefits also brought sadness and was seen as the end of future employment wishes and dreams:

“So when I finally was granted long term disability benefits, ‘disapproved for work’ as it is being called, I had a moment where I thought: Yes, disapproved, so is this it? […] So I noticed a vague feeling of sadness, like: yeah, this is the end of it.”

*(participant without paid employment).*

“These long term disability benefits are a sort of trap. See, they give me peace now [but] I have occasional moments where I think: perhaps I could do more, or I would like to apply for jobs. […] But if I would try […], and I would fail [..] then I have a problem [because I would loose my benefits].”

*(participant without paid employment)*

## Discussion

The aim of the present study was to explore the meaning of work for the subjective well-being of autistic adults with and without paid employment and to evaluate differences and similarities between the two groups. Generally, both groups viewed paid employment as highly important for their well-being, albeit for different reasons. Three themes were found, of which the first was: *Not having paid employment was associated with lacking societal recognition, and low self-esteem.* This was a major theme in the groups *without* paid employment and showed that not earning an income made participants feel unacknowledged and unvalued by others. Even if they had voluntary work, they often reported to feel guilty, ashamed, socially excluded, and this was associated with feelings of low self-esteem and low self-worth. Moreover, participants receiving long-term benefits talked about the ‘benefits trap’ they found themselves in: they were glad their financial stress had been solved, but fear of losing those benefits also stood in the way of their wishes for future re-employment. The second theme was: *Work can seriously damage health and well-being*. Most participants from both groups emphasized that work can be very damaging to health, and many participants had personally experienced this. Especially high stress levels and not seeing or respecting one’s own energy boundaries often had left participants depleted, burned out, and damaged. Even in the groups with paid employment, many participants had experienced one or more burnout episodes during their lives. In the groups without employment, the severe health damage resulting from previous employment experiences sometimes was reported as a reason why participants did not envision themselves ever working again, although even then they often could still talk about the positive sides of employment and elaborate on what their ideal job would look like. The third theme was: *Paid employment provides a variety of major benefits for well-being,* with subthemes: ‘purpose,’ ‘social contacts,’ ‘growth and use of talents,’ ‘structure and calmness’ and ‘income and freedom.’ A difference was that the groups with paid employment talked substantially longer and more elaborately about how the benefits of work positively affect well-being than the groups without.

A main finding of this study is that in both groups, the great majority of participants emphasized that paid employment was highly important to their well-being. Even if participants had experienced several damaging burnout periods during their lives or were on long-term disability benefits and did not have to pursue paid employment anymore, most of them still stressed the importance of having paid employment for their well-being. Most of the beneficial aspects of paid employment that participants mentioned as contributing to their well-being, are in line with findings from previous studies in both autistic and non-autistic populations, such as purpose, income and self-reliance, identity, social contacts and using talents, [e.g., 10, 24, 25, 29].

A new and important finding of the present study was that work can also provide a place of rest and calmness to buffer stress from family life, such as stress associated with social-emotional expectations from spouses or the hectic commotion of having young children. This finding is interesting, considering that autistic individuals have been found to often experience high stress levels in their lives [30, 31] and that burnout episodes from life stress seem to be common in this group [32, 33]. Although what has been described as ‘autistic burnout’ is conceptually different from how occupational burnout has been described in non-autistic working populations [33, 34], in both concepts it is a debilitating condition characterized by exhaustion. The Job Demands-Resources (JD-R) theory, a well-studied theory on occupational burnout, may therefore still be relevant for the prevention of autistic burnout [21]. Specifically, JD-R theory states that every job includes demands (i.e., ‘bad things’ that are subjectively experienced as costing negative energy) as well as job resources (‘good things’ that are enjoyable and give positive energy). Essentially, when job demands are chronically high and not compensated by job resources, employees’ energy is progressively drained, which can result in a state of mental exhaustion, burnout, and poor mental health [21, 34–36]. Hence, extending JD-R theory to the high life stress levels that autistic individuals can experience, this implies that if they can find positive energy resources to counterbalance the demands that cause negative stress, for instance by a job that provides rest or positive energy (as was shown from some quotes), this may well buffer against getting a burnout episode from life stress, and prevent from further damage and mental illness. Future research should study if the JD-R theory can be used to improve well-being and prevent mental illness in autistic adults.

Not earning one’s own income was associated with feelings of dependence, a lack of recognition from others and societal exclusion, even though these participants often held volunteer jobs. Receiving disability benefits was associated with feelings of low self-worth and self-esteem, shame, and uselessness. This is an important finding because this theme was so prominent, while the effects of receiving disability benefits on well-being are understudied. Several studies have shown that job loss and unemployment can be harmful to well-being, by diminishing self-esteem and result in loss of identity, social contacts, and complicated grief [37, 38]. However, results from the present study suggest it may especially be the aspect of not earning one’s own income that is harmful to well-being. Although the authors are not aware of any studies on autistic individuals where this was found, this finding is in line with a qualitative study on the well-being after involuntary job loss [39]. Here, it was shown that respondents experienced feelings of loss of dignity and belonging as a human being and felt insecurity and stress due to their changed financial situation, which in turn led to isolation and loss of self-esteem [39]. An explanation for the finding that receiving disability benefits was negatively associated with well-being may be found in the Capability Approach. According to this approach, well-being is determined by (a) *opportunities* individuals have, to be and do what they ‘value’ (find important), and (b) by real and unconstrained *freedom to make their own choices* about being and doing what they value [17, 40, 41]. Hence, the finding that well-being was negatively affected in many participants without paid employment may result from having neither much opportunity nor freedom to make choices concerning their employment situation. For instance, participants on long-term disability benefits felt that receiving benefits hindered their future re-employment dreams. It withheld them from being and doing what they found important for their well-being (being financially independent, finding a good and enjoyable job again).

The finding that participants on long-term disability benefits were ambivalent about receiving benefits, as it took their current financial stress away while simultaneously hindering their future re-employment dreams, is in line with findings of a review on the meaning of work to people with disabilities [24]. These authors described this phenomenon as a ‘benefits trap.’ In the present study, participants who were receiving disability benefits and who did not express any intention to take action for re-employment could often still describe what their ideal job would look like. However, there were two common reasons why participants did not tend to take action in trying to find paid employment. First, they feared losing their social benefits, and they regarded their financial security as very important for their well-being. Second, they were afraid that if their work reintegration would not be a success, it would lead to more damage to their mental health (e.g., burnout or depression). Therefore, the findings suggest that for successful work reintegration, they should be able to explore their talents and interests carefully and be able to gain positive work experiences without any financial pressure. Hence, to overcome the benefits trap, people need not having to fear a loss of income. Nevertheless, paid employment may not be the goal for everyone, and social welfare support should remain accessible for those who need it.

In both groups, most participants agreed that employment can be very damaging to health and well-being. Many participants had experienced one or multiple burnout episodes during their lives, which is in line with findings of others [32, 33], who argue that autistic burnout is a serious condition which is commonly described by autistic people but hardly recognized by the academic literature. Researchers have previously stated that autistic burnout is the result of an accumulation of chronic life stress, combined with a mismatch of expectations and abilities and no adequate supports [32]. Several studies have also shown that autistic individuals tend to have high life stress levels [30–32, 42] and employment may contribute to this, especially if there are problems at work, such as a lack of understanding and support [29]. Moreover, studies have shown that discontinuities in employment history are common for autistic adults [10, 43], which may be especially harmful for those who dislike interruptions and have difficulties starting and stopping activities [44]. Furthermore, the unemployment literature on the ‘Scarring’ effect has shown that (periods of) unemployment, but also of job insecurity and involuntary job loss, all continue to have lasting negative effects on mental health and well-being, even after people are re-employed [23, 45, 46]. Hence, autistic individuals who have discontinuous careers may experience even more stress than neurotypicals, because of previous negative experiences.

This study has several strengths and limitations. A first strength is that participants were recruited via different channels and mostly outside the mental health service system. Much of the current autism research has been criticized as it has too often recruited participants through the mental health service system [47]. Another strength is that this study evaluated and analyzed the views of people with and without work separately, taking into account important contextual circumstances that could be of influence on their views. Also, the study was initiated by an autistic junior researcher (MB), focuses on autistic well-being and acknowledges the important role of the context, and therefore adds to the much-needed body of knowledge on autistic flourishing [48]. Concerning limitations, the study population only entailed Caucasian and relatively highly educated participants and limited information was collected on the types of employment they had. Moreover, it may be that especially people who value paid employment as important for their well-being were interested in participating in the study, biasing the results. Another limitation is that the focus groups participants were selected on the basis of their employment status at the time of the study, neither taking into account their employment history, nor whether they were feeling and performing well at work. Finally, the fact that the theme regarding the benefits of work had five subthemes, while the other themes had none may have been caused by the topic of our study (‘The meaning of employment for well-being’), as the use of the word ‘meaning’ may tend to elicit more positive reflections than negative ones. Whether or not caused by the term ‘meaning,’ the positive meaning of work was discussed most elaborately, and longer discussions can lead to more subthemes in qualitative research. This underlines the importance to further study the themes ‘lack of societal recognition’ and the ‘harmful effects of paid work for mental health’ in future research.

In conclusion, the findings from both groups strongly highlight the importance of paid employment for the well-being of autistic adults, albeit for different reasons. However, both also agreed that paid employment can be very harmful to (mental) health and well-being. Future research should focus on how to prevent autistic burnout and to reduce life and occupational stress levels for autistic adults. A suitable, well-supported job may help to buffer stress in other life areas. More studies are needed on how healthy jobs can be created where autistic adults get positive energy and experience high well-being. This is also important to reduce socio-economic inequalities.

## Supplementary Information

Below is the link to the electronic supplementary material.

Supplementary file1 (PDF 418 kb)

## Data Availability

The datasets generated during the current study are available from the corresponding author on reasonable request.

## Footnotes

1 We use identity-first language (‘autistic person’) but respect that some prefer person-first language (‘person with autism’), e.g., see [1, 2].
